# Supplementary material for: Omicron SARS-CoV-2 epidemic in England during February 2022: A series of cross-sectional community surveys
Source: Lancet Reg Health Eur. 2022 Jul 28;21:100462. doi: 10.1016/j.lanepe.2022.100462 (PMC9330654; doi:10.1016/j.lanepe.2022.100462)
Supplement: Supplementary file 1 [file mmc1.pdf]

Supplementary Information:

Cross-sectional community surveys to monitor the Omicron SARS-CoV-2 epidemic in England during February 2022.

Short Title: Omicron SARS-CoV-2 sublineages in England

Marc Chadeau-Hyam[1,2,*], David Tang[1,2**], Oliver Eales[1,3,**], Barbara Bodinier[1,2,**], Haowei Wang[1,3], Jakob Jonnerby[1,3], Matthew Whitaker[1,2], Joshua Elliott[4,5], David Haw[1,3], Caroline E. Walters[1,3], Christina Atchison[1], Peter J. Diggle[6], Andrew J. Page[7], Deborah Ashby[1], Wendy Barclay[4], Graham Taylor[4], Graham Cooke[4,5,8], Helen Ward[1,5,8], Ara Darzi[5,8,9], Christl A. Donnelly[1,3,10,†,*], Paul Elliott[1,2,5,8,11,12,†*]

[1] School of Public Health, Imperial College London, UK
[2] MRC Centre for Environment and Health, School of Public Health, Imperial College London, UK
[3] MRC Centre for Global infectious Disease Analysis and Jameel Institute, Imperial College London, UK
[4] Department of Infectious Disease, Imperial College London, UK
[5] Imperial College Healthcare NHS Trust, UK
[6] CHICAS, Lancaster Medical School, Lancaster University, UK and Health Data Research, UK
[7] Quadram Institute, Norwich, UK
[8] National Institute for Health Research Imperial Biomedical Research Centre, UK
[9] Institute of Global Health Innovation, Imperial College London, UK
[10] Department of Statistics, University of Oxford, UK
[11] Health Data Research (HDR) UK, Imperial College London, UK
[12] UK Dementia Research Institute, Imperial College London, UK

**Equal contribution
†Joint last authors
*Corresponding authors: Paul Elliott, Christl A Donnelly, and Marc Chadeau-Hyam
p.elliott@imperial.ac.uk, c.donnelly@imperial.ac.uk, m.chadeau@imperial.ac.uk, School of Public Health, Imperial College London, Norfolk Place, London, W2 1PG

Keywords: COVID-19, SARS-CoV-2, Omicron variant, BA.2 sublineage, random community surveys

Table S1. Unweighted and weighted prevalence of swab-positivity from REACT-1 across rounds 1 to 18.

Round	Tested swabs	Positive swabs	Unweighted prevalence (95% CI)	Weighted prevalence (95% CI)	First sample	Last sample
1	120,620	159	0.13% (0.11%, 0.15%)	0.16% (0.13%, 0.19%)	01/05/20	01/06/20
2	159,199	123	0.08% (0.07%, 0.09%)	0.09% (0.07%, 0.11%)	19/06/20	07/07/20
3	162,821	54	0.03% (0.03%, 0.04%)	0.04% (0.03%, 0.05%)	24/07/20	11/08/20
4	154,325	137	0.09% (0.08%, 0.11%)	0.13% (0.01%, 0.15%)	20/08/20	08/09/20
5	174,949	824	0.47% (0.44%, 0.50%)	0.60% (0.55%, 0.71%)	18/09/20	05/10/20
6	160,175	1,732	1.08% (1.03%, 1.13%)	1.30% (1.21%, 1.39%)	16/10/20	02/11/20
7	168,181	1,299	0.77% (0.73%, 0.82%)	0.94% (0.87%, 1.01%)	13/11/20	03/12/20
8	167,642	2,282	1.36% (1.31%, 1.42%)	1.57% (1.49%, 1.66%)	06/01/21	22/01/21
9	165,456	689	0.42% (0.39%, 0.45%)	0.49% (0.44%, 0.55%)	04/02/21	23/02/21
10	140,844	227	0.16% (0.14%, 0.18%)	0.20% (0.17%, 0.23%)	11/03/21	30/03/21
11	127,408	115	0.09% (0.07%, 0.11%)	0.10% (0.08%, 0.13%)	15/04/21	03/05/21
12*	108,911	135	0.12% (0.10%, 0.15%)	0.15% (0.12%, 0.18%)	20/05/21	07/06/21
13	98,233	527	0.54% (0.49%, 0.58%)	0.63% (0.57%, 0.69%)	24/06/21	12/07/21
14**	100,527	764	0.76% (0.71%, 0.82%)	0.83% (0.76%, 0.89%)	09/09/21	27/09/21
15***	100,112	1,399	1.40% (1.33%, 1.47%)	1.57% (1.48%, 1.66%)	19/10/21	05/11/21
16****	97,089	1,192	1.23% (1.16%, 1.30%)	1.41% (1.33%, 1.51%)	23/11/21	14/12/21
17†	102,174	4,073	3.99% (3.87%, 4.11%)	4.41% (4.25%, 4.56%)	05/01/22	20/01/22
18‡	94,950	2,731	2.88% (2.77%, 2.98%)	2.88% (2.76%, 3.00%)	08/02/22	01/03/22

* Sampling strategy changed for round 12 and subsequent rounds. Therefore unweighted prevalence is not directly comparable with previous rounds

** Including N=509 samples from 28-30 September 2021. Sample handling changed in round 14. Therefore prevalence is not directly comparable with previous rounds

*** Including N=93 samples (all negatives) from 6-8 November 2021, and N=86 samples with no collection/arrival dates

**** Including N=661 samples (including 12 positives) from 15-17 December 2021. Swab positivity was assessed using a multiplex assay from round 16 onwards. Test diagnostic characteristics may differ slightly from previous rounds

† Including N=862 (including 36 positives) from 21-24 January 2022

‡ Including N=685 (including 18 positives) from 2-4 March 2022

Table S2. Table of growth rates, reproduction numbers and doubling/halving times from exponential model fits on data from round 17 (5 to 20 January 2022) and round 18 (8 February to 1 March 2022)

Rounds			Growth rate per day (r)	Reproduction number (R)**	Probability R>1, r>0	Doubling (+) / Halving (-) time (in days)
18	All positives		-0.019 (-0.026 , -0.012)	0.94 (0.91 , 0.96)	<0.01	-36.6 (-26.5 , *)
	Age	Aged 17 and under	-0.063 (-0.079 , -0.047)	0.79 (0.74 , 0.84)	<0.01	-11.0 (-8.8 , -14.7)
		Aged 18 to 54	-0.023 (-0.033 , -0.013)	0.92 (0.89 , 0.96)	<0.01	-30.0 (-21.0 , *)
		Aged 55 and over	0.013 (-0.001 , 0.027)	1.04 (1.00 , 1.09)	0.96	* (* , 25.5)
	Region	East Midlands	-0.040 (-0.068 , -0.013)	0.87 (0.77 , 0.96)	<0.01	-17.4 (-10.2 , *)
		West Midlands	-0.040 (-0.065 , -0.016)	0.87 (0.78 , 0.95)	<0.01	-17.5 (-10.7 , -44.6)
		East of England	-0.026 (-0.047 , -0.005)	0.91 (0.84 , 0.98)	0.01	-26.8 (-14.7 , *)
		London	-0.020 (-0.036 , -0.003)	0.94 (0.88 , 0.99)	0.01	-35.5 (-19.1 , *)
		North West	-0.005 (-0.026 , 0.016)	0.98 (0.91 , 1.05)	0.32	* (-26.5 , 42.4)
		North East	0.007 (-0.030 , 0.042)	1.02 (0.90 , 1.14)	0.64	* (-23.0 , 16.3)
		South East	-0.021 (-0.038 , -0.005)	0.93 (0.87 , 0.98)	0.01	-32.8 (-18.4 , *)
		South West	-0.018 (-0.041 , 0.004)	0.94 (0.86 , 1.01)	0.05	-38.1 (-17.0 , *)
		Yorkshire and The Humber	-0.007 (-0.032 , 0.018)	0.98 (0.89 , 1.06)	0.30	* (-21.9 , 38.2)
17 - 18	All positives		-0.012 (-0.014 , -0.011)	0.96 (0.96 , 0.96)	<0.01	* (* , *)
	Age	Aged 17 and under	-0.013 (-0.016 , -0.011)	0.96 (0.95 , 0.97)	<0.01	* (-43.5 , *)
		Aged 18 to 54	-0.013 (-0.015 , -0.011)	0.96 (0.95 , 0.96)	<0.01	* (-46.6 , *)
		Aged 55 and over	-0.011 (-0.013 , -0.008)	0.96 (0.96 , 0.97)	<0.01	* (* , *)
	Region	East Midlands	-0.015 (-0.019 , -0.010)	0.95 (0.94 , 0.97)	<0.01	-47.6 (-36.0 , *)
		West Midlands	-0.019 (-0.023 , -0.015)	0.94 (0.92 , 0.95)	<0.01	-36.7 (-30.2 , -46.7)
		East of England	-0.005 (-0.009 , -0.001)	0.98 (0.97 , 1.00)	0.01	* (* , *)
		London	-0.012 (-0.015 , -0.009)	0.96 (0.95 , 0.97)	<0.01	* (-45.5 , *)
		North West	-0.021 (-0.024 , -0.017)	0.93 (0.92 , 0.94)	<0.01	-33.5 (-28.4 , -40.6)
		North East	-0.029 (-0.035 , -0.023)	0.90 (0.88 , 0.92)	<0.01	-24.1 (-19.9 , -30.3)
		South East	0.000 (-0.004 , 0.003)	1.00 (0.99 , 1.01)	0.41	* (* , *)
		South West	0.001 (-0.003 , 0.005)	1.00 (0.99 , 1.02)	0.67	* (* , *)
		Yorkshire and The Humber	-0.023 (-0.027 , -0.019)	0.92 (0.91 , 0.94)	<0.01	-30.1 (-25.3 , -36.8)

* Doubling/Halving time had an estimated magnitude greater than 50 days and so represented approximately constant prevalence

** Within-round R was calculated assuming an Omicron-specific Gamma-distributed generation time with mean 3.3 days and standard deviation of 3.5 days. The equation used in estimating R from r is only valid for r>-0.27 (the rate parameter of the Gamma distribution) where this is not the case there is an NA

Table S3A. Weighted prevalence of SARS-CoV-2 swab-positivity in round 17 and round 18 by sex, age, region, urban status, employment type, and ethnic group.

		Round 17			Round 18		
Variable		Positive	Total	Weighted Prevalence*	Positive	Total	Weighted Prevalence*
Sex	Male	1,858	45,031	4.52% (4.29%, 4.76%)	1,199	40,936	2.97% (2.80%, 3.15%)
	Female	2,215	57,141	4.30% (4.11%, 4.50%)	1,532	54,005	2.79% (2.64%, 2.95%)
	Unknown	0	2	**	0	9	**
Age	05-11	396	5,287	7.85% (7.10%, 8.69%)	161	3,392	4.69% (4.01%, 5.48%)
	12-17	255	5,351	5.20% (4.57%, 5.92%)	261	7,584	3.42% (3.00%, 3.91%)
	18-24	132	2,649	4.96% (4.11%, 5.96%)	205	7,407	2.70% (2.33%, 3.13%)
	25-34	392	7,683	5.08% (4.57%, 5.65%)	463	14,970	3.05% (2.77%, 3.35%)
	35-44	599	12,348	5.02% (4.62%, 5.46%)	492	14,386	3.43% (3.13%, 3.76%)
	45-54	678	16,996	4.05% (3.74%, 4.38%)	371	11,976	3.09% (2.79%, 3.43%)
	55-64	777	21,590	3.71% (3.45%, 3.99%)	343	14,405	2.37% (2.13%, 2.64%)
	65-74	597	20,088	3.06% (2.82%, 3.33%)	308	13,635	2.21% (1.98%, 2.48%)
	75+	247	10,182	2.46% (2.16%, 2.80%)	127	7,195	1.68% (1.41%, 2.01%)
Region	South East	545	18,287	3.23% (2.94%, 3.55%)	514	15,978	3.33% (3.04%, 3.66%)
	North East	273	4,541	6.86% (5.99%, 7.84%)	100	4,364	2.33% (1.88%, 2.87%)
	North West	589	11,867	5.36% (4.88%, 5.88%)	291	11,386	2.58% (2.28%, 2.92%)
	Yorkshire and The Humber	488	9,736	5.53% (4.98%, 6.13%)	229	9,133	2.48% (2.15%, 2.85%)
	East Midlands	352	9,045	4.15% (3.69%, 4.67%)	211	8,295	2.53% (2.18%, 2.93%)
	West Midlands	476	10,297	5.20% (4.70%, 5.75%)	264	9,745	2.63% (2.31%, 2.99%)
	East of England	350	11,938	3.42% (3.05%, 3.83%)	298	10,658	2.92% (2.58%, 3.29%)
	London	682	14,849	4.89% (4.49%, 5.31%)	508	15,480	3.20% (2.92%, 3.52%)
	South West	318	11,614	2.92% (2.58%, 3.30%)	316	9,911	3.12% (2.77%, 3.51%)
Living in urban area	Yes	3,402	79,566	4.72% (4.54%, 4.90%)	2,217	76,453	2.92% (2.80%, 3.06%)
	No	661	22,405	3.14% (2.89%, 3.42%)	501	18,249	2.68% (2.44%, 2.94%)
	Unknown	10	203	6.07% (2.99%, 11.92%)	13	248	3.90% (2.22%, 6.75%)
Employment type	Health care or care home worker	379	7,966	5.31% (4.75%, 5.94%)	269	7,812	3.27% (2.87%, 3.73%)
	Other essential/key worker	722	14,170	5.39% (4.97%, 5.84%)	418	13,396	3.14% (2.83%, 3.48%)
	Other worker	1,586	38,328	4.57% (4.32%, 4.83%)	1,093	37,420	3.01% (2.83%, 3.21%)
	Not full-time, part-time, or self-employed	1,281	39,792	3.52% (3.30%, 3.75%)	885	34,073	2.54% (2.36%, 2.72%)
	Unknown	105	1,918	5.70% (4.63%, 7.00%)	66	2,249	2.79% (2.16%, 3.60%)
Ethnic group	White	3,421	89,773	4.13% (3.98%, 4.28%)	2,346	80,897	2.90% (2.78%, 3.03%)
	Asian	297	5,383	6.58% (5.76%, 7.50%)	194	6,642	2.95% (2.52%, 3.43%)
	Black	105	1,730	6.55% (5.33%, 8.01%)	44	2,063	2.27% (1.66%, 3.10%)
	Mixed	91	1,745	5.56% (4.48%, 6.88%)	63	1,997	3.29% (2.50%, 4.31%)
	Other	58	963	7.35% (5.57%, 9.63%)	29	1,083	2.94% (1.97%, 4.36%)
	Unknown	101	2,580	4.32% (3.48%, 5.34%)	55	2,268	2.27% (1.70%, 3.02%)

* Due to large numbers of missing reported dates of past infection, prevalence estimates are based on the whole study population and do not account for potential prior immunity

** Prevalence estimates are not reported if based on less than 10 observations

Table S3B. Weighted prevalence of SARS-CoV-2 swab-positivity in round 17 and round 18 by household size, number of children in the household, contact with a COVID-19 case, protective behaviours, symptom status and neighbourhood deprivation

		Round 17			Round 18		
Variable		Positive	Total	Weighted Prevalence	Positive	Total	Weighted Prevalence
Household size	1	536	17,601	3.15% (2.86%, 3.48%)	288	14,239	1.87% (1.65%, 2.11%)
	2	1,304	40,719	3.38% (3.19%, 3.59%)	845	33,450	2.50% (2.33%, 2.68%)
	3	786	17,384	4.95% (4.58%, 5.36%)	543	18,194	3.06% (2.79%, 3.35%)
	4	941	18,422	5.41% (5.04%, 5.80%)	716	19,042	3.88% (3.58%, 4.20%)
	5	337	5,719	6.23% (5.52%, 7.01%)	219	6,570	3.33% (2.87%, 3.85%)
	6+	169	2,329	7.72% (6.47%, 9.18%)	120	3,455	3.36% (2.76%, 4.09%)
Number of children in the household	0	2,306	68,713	3.57% (3.41%, 3.74%)	1,407	58,734	2.31% (2.19%, 2.44%)
	1+	1,535	28,206	5.92% (5.60%, 6.25%)	1,072	28,474	3.88% (3.63%, 4.14%)
	Unknown	232	5,255	4.75% (4.14%, 5.45%)	252	7,742	3.16% (2.78%, 3.60%)
COVID case contact	No	1,605	75,859	2.40% (2.27%, 2.54%)	1,187	69,708	1.73% (1.62%, 1.84%)
	Yes, contact with a confirmed/tested COVID-19 case	1,781	13,692	12.83% (12.20%, 13.49%)	1,056	10,312	10.35% (9.72%, 11.02%)
	Yes, contact with a suspected COVID-19 case	230	2,562	9.38% (8.14%, 10.79%)	142	2,098	6.86% (5.77%, 8.15%)
	Unknown	457	10,061	5.30% (4.78%, 5.86%)	346	12,832	2.55% (2.27%, 2.85%)
Shielding	Yes	2,809	26,223	3.44% (3.18%, 3.73%)	426	17,277	2.39% (2.16%, 2.65%)
	No	801	65,800	4.60% (4.41%, 4.80%)	1,953	64,720	3.08% (2.93%, 3.23%)
	Unknown	463	10,151	5.31% (4.80%, 5.88%)	352	12,953	2.58% (2.31%, 2.89%)
Frequency wearing mask indoors	Always	1,774	49,795	3.84% (3.64%, 4.05%)	717	24,303	2.85% (2.64%, 3.08%)
	Sometimes	1,295	31,768	4.33% (4.07%, 4.61%)	1,111	37,147	3.01% (2.83%, 3.20%)
	Hardly ever	131	2,695	5.22% (4.32%, 6.30%)	109	3,901	2.89% (2.37%, 3.52%)
	Never	68	1,640	4.60% (3.43%, 6.15%)	36	1,378	2.40% (1.70%, 3.40%)
	Unknown	805	16,276	5.77% (5.35%, 6.22%)	758	28,221	2.77% (2.56%, 3.00%)
Symptom status	Classic COVID symptoms**	1,673	10,481	15.85% (15.06%, 16.68%)	1,175	7,845	15.06% (14.21%, 15.95%)
	Other symptoms	930	16,525	5.81% (5.40%, 6.24%)	530	13,651	3.85% (3.52%, 4.22%)
	No symptoms	1,019	65,149	1.87% (1.74%, 2.01%)	682	60,676	1.17% (1.08%, 1.27%)
	Unknown	451	10,019	5.26% (4.74%, 5.82%)	344	12,778	2.53% (2.26%, 2.84%)
Deprivation	1 Most deprived	639	11,245	6.08% (5.58%, 6.61%)	366	13,161	2.69% (2.41%, 3.00%)
	2	763	17,276	4.62% (4.27%, 4.99%)	458	17,218	2.68% (2.42%, 2.95%)
	3	831	21,472	3.96% (3.68%, 4.26%)	597	19,829	2.98% (2.73%, 3.24%)
	4	895	24,536	3.93% (3.66%, 4.22%)	644	21,549	3.13% (2.88%, 3.40%)
	5 Least deprived	945	27,645	3.69% (3.44%, 3.96%)	666	23,193	2.90% (2.67%, 3.14%)

* Due to large numbers of missing reported dates of past infection, prevalence estimates are based on the whole study population and do not account for potential prior immunity

** Classic COVID symptoms: loss or change of sense of smell or taste, fever, new persistent cough

Table S4. Multivariable logistic regression for round 18. Results are presented as Odds Ratios (95% confidence interval) adjusted for age and sex and additionally for region and all other variables (mutually adjusted OR).

	Category	Round 18	
		Adjusted for age and sex*	Mutually adjusted*,**
Sex	Male	Ref	Ref
	Female	0.96 (0.88, 1.03)	0.94 (0.87, 1.01)
Employment type	Health care or care home worker	1.15 (1.01, 1.32)	1.20 (1.05, 1.38)
	Other essential/key worker	1.03 (0.92, 1.15)	1.04 (0.92, 1.16)
	Other worker	Ref	Ref
	Not full-time, part-time, or self-employed	1.00 (0.90, 1.11)	1.02 (0.92, 1.14)
	Unknown	0.91 (0.70, 1.18)	0.94 (0.72, 1.22)
Ethnic group	White	Ref	Ref
	Asian	0.90 (0.77, 1.05)	0.84 (0.72, 0.98)
	Black	0.66 (0.49, 0.89)	0.60 (0.44, 0.82)
	Mixed	0.93 (0.72, 1.20)	0.90 (0.70, 1.16)
	Other	0.84 (0.58, 1.22)	0.79 (0.54, 1.15)
	Unknown	0.85 (0.65, 1.12)	0.83 (0.63, 1.09)
Household size	1-2	Ref	Ref
	3-5	1.22 (1.11, 1.34)	1.07 (0.95, 1.19)
	6+	1.26 (1.03, 1.54)	1.12 (0.90, 1.39)
Number of children in the household	0	Ref	Ref
	1+	1.36 (1.23, 1.50)	1.33 (1.18, 1.50)
	Unknown	1.15 (0.89, 1.49)	1.17 (0.90, 1.52)
Living in urban area	Yes	1.00 (0.91, 1.11)	1.03 (0.93, 1.14)
	No	Ref	Ref
	Unknown	1.74 (0.99, 3.07)	1.82 (1.03, 3.21)
Deprivation	1 Most deprived	0.90 (0.79, 1.03)	0.99 (0.87, 1.14)
	2	0.89 (0.78, 1.00)	0.92 (0.81, 1.04)
	3	1.03 (0.92, 1.15)	1.05 (0.94, 1.18)
	4	1.03 (0.93, 1.16)	1.05 (0.94, 1.18)
	5 Least deprived	Ref	Ref

*Odds ratios are not adjusted for possible prior immunity

**Odds ratios are mutually adjusted for all variables shown and for age and region
